# Cognition and chronic hypoxia in pulmonary diseases

**DOI:** 10.1590/S1980-57642010DN40100003

**Published:** 2010

**Authors:** Renata Areza-Fegyveres, Ronaldo A. Kairalla, Carlos R.R. Carvalho, Ricardo Nitrini

**Affiliations:** 1 Neurologist, collaborating researcher of the Cognitive and Behavioral Neurology Unit, Hospital das Clínicas, University of São Paulo Medical School.; 2 Assistant Professor, Pulmonary Division, Heart Institute (InCor), University of São Paulo Medical School.; 3 Associate Professor, Pulmonary Division, Heart Institute (InCor), University of São Paulo Medical School.; 4 Associate Professor of the Department of Neurology and Director of the Cognitive and Behavioral Neurology Unit, Hospital das Clínicas, University of São Paulo Medical School.

**Keywords:** chronic hypoxia, brain, cognitive impairment, neuropsychological tests, encephalopathy of the subcortical type

## Abstract

Lung disease with chronic hypoxia has been associated with cognitive impairment of the subcortical type.

**Objectives:**

To review the cognitive effects of chronic hypoxia in patients with lung disease and its pathophysiology in brain metabolism.

**Methods:**

A literature search of Pubmed data was performed. The words and expressions from the text subitems including “pathophysiology of brain hypoxia”, “neuropsychology and hypoxia”, “white matter injury and chronic hypoxia”, for instance, were key words in a search of reports spanning from 1957 to 2009. Original articles were included.

**Results:**

According to national and international literature, patients with chronic obstructive pulmonary disease and sleep obstructive apnea syndrome perform worse on tests of attention, executive functions and mental speed. The severity of pulmonary disease correlates with degree of cognitive impairment. These findings support the diagnosis of subcortical type encephalopathy.

**Conclusion:**

Cognitive effects of clinical diseases are given limited importance in congresses and symposia about cognitive impairment and its etiology. Professionals that deal with patients presenting cognitive loss should be aware of the etiologies outlined above as a major cause or potential contributory factors, and of their implications for treatment adherence and quality of life.

## Abstract (pt)

As doenças pulmonares que cursam com hipóxia crônica tem sido associadas à alteração cognitiva do tipo subcortical.

**Objetivo:**

Revisar os efeitos cognitivos da hipóxia crônica em pacientes com doenças pulmonar e sua fisiopatologia.

**Métodos:**

Foi utilizado o banco de dados do Pubmed. As palavras e expressões foram os temas dos subitens da revisão como, por exemplo, “fisiopatologia e hipóxia cerebral”, “neuropsicologia e hipoxia”, “lesões de substância branca e hipóxia crônica”, variando de 1957 to 2009. Artigos originais foram incluídos.

**Resultados:**

De acordo com a literatura nacional e internacional, pacientes com doença pulmonar obstrutiva crônica e síndrome da apnéia obstrutiva do sono apresentam desempenho pior em testes neuropsicológicos que avaliam atenção, funções executivas e velocidade de processamento mental. Esses achados configuram uma encefalopatia do tipo subcortical.

**Conclusion:**

É dada importância limitada às conseqüências cognitivas das doenças clínicas em congressos e simpósios sobre cognição e suas etiologias. Profissionais que lidam com pacientes que apresentam perda cognitiva devem suspeitar das etiologias mencionadas acima com causa principal ou como co-fatores, assim com suas implicações na aderência ao tratamento e qualidade de vida.

There is a delicate balance between functioning of the central nervous system (CNS) and the ventilatory system.^1^ Slight changes can have a significant impact.^1,2^ Acute or chronic respiratory insufficiency can result in a myriad of neurological and neuropsychological signs and symptoms which are ultimately consequences of hypoxia and hypercapnia.

Cardiac, pulmonary and hematological diseases can cause hypoxia. Hypoxia can also manifest in specific situations such as in aircraft travel and high altitude climbing. Hypoxia brain effects depend on the severity, duration, speed of onset and progression of the condition. Thus, patients with chronic hypoxia will present different findings from those with acute respiratory distress.^1,2^ In addition, patients with compromised respiratory control or neuromuscular disease can hypoventilate, thereby enhancing the carbon dioxide partial pressure (PaCO2).

Initially, descriptions of neurological and behavioral findings concerning respiratory disease were available for end-stage disease. These included papilloedema and loss of visual acuity,^3^ intracranial hypertension,^4^ headache, somnolence, tremor and *asterix*.^5^ Irritability, anxiety, mental confusion and psychotic symptoms were also reported^6,7^ at more advances stages.

Original articles published up to 2009 were searched on the Pubmed database. The following words and expressions were used as key search terms, alone or together: “chronic hypoxia”, “pathophysiology”, “neuropsychological tests”, “brain”, “white matter lesions”, “pulmonary disease”, “lung disease”, “cognition”, “dementia”, “cognitive impairment”. More than 300 articles were found. Search results were screened for content and historical relevance of each subitem.

## Pathophysiology of chronic hypoxia effects in central nervous system metabolism

Hypoxia is a widely used term but ideally, it should be previously defined. In most studies the term means oxygen levels which are below oxygen atmospheric concentration. This can occur when the inspired oxygen concentration is low, thus resulting in “hypoxaemic hypoxia” or when the general barometric pressure is low, called “hypobaric hypoxia” a situation naturally produced when climbing at high altitudes. There is no evidence of significant difference in adaptative response mechanisms between the two previously mentioned settings or methods of producing continuous exposition to chronic hypoxia.

Severity of hypoxia is often ill-defined. The majority of investigators refer to three severity levels: mild, moderate and severe, but no consensus exists on the boundaries between levels. Most consider mild stage as when oxygen partial pressure (PaO2) is above 50 mmHg, assuming normal red blood cell volume. At this level, there is complete compensation and general function is barely altered. The equivalent of ten percent of normobaric oxygen concentration, or 5000 meters of altitude, is the upper limit of mild hypoxia. Oxygen partial pressure between 35 and 50 mmHg is generally considered moderate hypoxia, a state which leads to variable findings in cognition. When Pa O2 is below 35 mmHg, there is loss of conscience. Moderate and severe hypoxia can result in variable neuronal loss according to severity and length of exposition.^5^

In the majority of studies, the expression “chronic hypoxia” was vague and usually corresponded to the interval of time necessary to trigger a physiologic response, which can vary from weeks to months.^8^ Thus, the definition of chronic hypoxia to describe constantly low oxygen (O2) saturation levels warrants comment. Some studies describing chronic hypoxia involved patients that were not hypoxaemic based on pulse oxymetry. In fact, these patients frequently presented periods of hypoxia, especially when exercising,^9,10^ during activities of daily living^11^ and sleep.^12,13^ Although the use of the expression “chronic hypoxia” is accepted in chronic obstructive pulmonary disease (COPD) for instance, its timely measurement during evaluation can yield results which fall between normal limits. The expression will be used in a consistent way throughout this manuscript.

The majority of encephalic neurons are “sensitive” to plasmatic oxygen concentration levels. They modify their activity in response to hypoxia lowering their metabolic rate and thus, reduce the production of adenosine triphosphate through oxidative phosphorylation. The major metabolic cost is to maintain the ionic gradient, which is directly associated with neuronal activity levels. However, not all neurons diminish their activities during hypoxia. There are special populations of neurons that act similarly to oxygen chemoreceptors. These oxygen “sensors” in the CNS monitor brain oxygen levels and when “active”, trigger critical processes necessary for the functioning of the organism. These chemoreceptors play a critical role in both short and long-term hypoxia adaptation mechanisms.

Survival after exposition to hypoxia is essentially associated to changes related to cardiovascular and respiratory systems in order to maintain oxygen delivery to tissues. In the CNS, the sites responsible for controlling sympathetic and respiratory activities are the thalamus, hypothalamus, pons and medulla.^14-17^ The “activation” of neurons in these areas produces enhancement of respiratory and sympathetic activities.^17^

The mechanism for detecting hypoxia and generating an adaptive response is governed by length of exposition: acute (for instance, hypoxic-ischaemic encephalopathy and acute respiratory insufficiency), subacute or chronic (for instance, high altitudes and COPD) and intermittent (obstructive sleep apnea syndrome - OSAS). The physiologic responses to hypoxia probably reflect changes in ionic channels, oxygen “sensors” (for example, heme proteins), signaling pathways, neuromodulators and genomic processes:^18-20^

### Ion channels

Hypoxia triggers depolarization of potassium, calcium and sodium channels leading to higher cell excitability. Hypoxia also reduces potassium ions in carotid glomus cells resulting in depolarization and opening of voltage-dependent calcium ion channels. This is followed by enhancement of intracellular calcium and activation of sensitive afferent nerves.

However, the effects of chronic hypoxia on ion channels activity are variable. The presence or absence of neurotrophic factors might be important in explaining the different effects of chronic hypoxia as the upregulation of sodium ion channels can depend on these factors, all of which could worsen hypoxia.^21,22^

### Oxygen sensitivity adaptation

Peripheral and CNS sensors adapt to sustained or chronic hypoxia. The respiratory and sympathetic responses to chronic or intermittent hypoxia are the final result of a cascade of adaptation events. The short-term response to sustained hypoxia is reduced respiration, followed by enhancement of sympathetic and respiratory activities which can be sustained for days or years. If hypoxia is intermittent, variable degrees of adaptative response occurs depending on the frequency and/or degree of hypoxia. Apparently, oxygen-sensitive neurons adapt to chronic or sustained hypoxia because their sensitivity rises after four or five days under these conditions.^23^ The nature of these changes involves modification in signaling pathways, in neuromodulators and their receptors (opioids, nitric oxide, P substance, catecholamines, glutamate and gama-aminobutyric acid) and in the genomic effects. This latter effect is followed by up and downregulation of the product generated by hypoxia-sensitive genes.

### Vascular mechanisms

The relationship between brain function and blood flow has been studied since the publication of Roy and Sherrington (1890 *apud* 24) in the late 19^th^ century. The first quantitative study^25^ showed a rise and then fall in cerebral blood flow (CBF) in healthy volunteers that initially breathed atmospheric air at sea level. Subsequently, they were transferred to a 3810m altitude laboratory in California, returning afterwards to sea level. However, many aspects of CBF control remain unknown.

### Vascular adaptations to chronic hypoxia

Reduced oxygen delivery is considered the environmental trigger to activate adaptative responses. Nevertheless, the contribution of each variable to the control mechanism has yet to be determined. Regarding systemic circulation, the primary variable is PaO2. The second is hemoglobin concentration level (oxygen carrier) in red blood cells, measured in milligrams per deciliter or by the hematocrit. The third factor is the hemoglobin saturation curve that is altered by temperature, pH, PaCO2 and 2,3 diphosphoglicerate. In the CNS, both CBF and capillary density (intercapilar distance) play critical roles.

**Cerebral blood flow (CBF):** Mild hypoxia augments CBF almost two-fold and lowers PaCO2 (26-29). The exact mechanism is unknown, but there is a main neurogenic component originating from the brain stem (30). Local signaling substances also influence CBF, for instance, vasodilator nitric oxide up or downregulates according to oxi-hemoglobin fall. Local tissue factors are more associated to intracerebral circulation distribution than to blood flow of the whole organism. Potassium ions, adenosine, nitric oxide and other substances play a secondary role and become more important as the hypoxia becomes more severe (31). The main mechanism responsible for at least half of the CBF rise in response to mild hypoxia is mediated through neuronal pathways that cross or originate in the brain stem^32-34^ and are closely linked to blood oxygen concentration levels.^35-36^

When hypoxia exposition is prolonged for more than one day, CBF is attenuated.^37.38^ After three weeks of sustained hypoxia CBF returns to previous levels.

**Hematocrit:** One of the main reasons for the return of CBF to previous levels is the rise in red cell volume.^39^ The oxygen content is compensated by the enhancement of its carrier, leading to pre-hypoxia status of oxygen delivery.

**Angiogenesis and brain blood volume:** Although oxygen delivery to the CNS is relatively compensated after exposition to chronic hypoxia, the same does not occur in the mitochondria. There is a reduction in oxygen delivery to the tissues because the stream that guides oxygen diffusion from the capillaries to the tissues is the difference between PaO2 of both of these. Consequently, there is a progressive rise in capillary density throughout angiogenesis that is complete after three weeks of hypoxia exposition.^37,40-42^

Angiogenesis occurs through hypoxia-inducible factor-1 which also leads to the enhancement of erythropoietin and hematocrit. Hypoxia-inducible Factor 1 upregulates the production of endothelial vascular growth factor. Angiopoietin-2-cicloxygenase-2 also contributes to brain angiogenesis.^43^

**Tissue oxygen tension:** The oxygen tissue tension is low and its distribution is heterogeneous even in normoxia conditions.^44,45^ The response time is variable: the CBF rises promptly and falls on the fourth or fifth day.^38^ The hematocrit begins to rise on the third day and reaches 80% within seven days. Angiopoetin-2 rises in the second week and subsequently falls to previous levels within three weeks.^42^ The hypoxia-inducible factor-1 which indicates tissue hypoxia is elevated initially and followed by a drop to previous levels within three weeks.^46^ These data show that the restoration of brain tissue oxygen tension does not occur until two or three weeks after hypoxia exposition.

**Average transit time:** The return of CBF to previous levels does not mean that cerebral circulation has not gone through significant changes. Brain blood flow and volume are directly related. If cerebral blood volume duplicates after hypoxia adaptation, the average transit time enhances considerably. This means that glucose delivery time is also elevated. The effect of improved glucose delivery is evidenced by better glucose influx through the hematoencephalic barrier after chronic hypoxia adaptation.^47^ There is an enhancement in the number of glucose transporter molecules per microvase besides a rise in capillary density. Findings of studies in humans are generally similar to those involving other mammals.^48^

## Cognitive impairment in pulmonary diseases with chronic hypoxia

In recent decades, several studies have demonstrated the presence of cognitive impairment caused by mild to moderate hypoxia and/or hypercarbia in patients with COPD,^49-75^ OSAS,^76-84^ subjects exposed to artificially induced hypoxia^85,86^ and high altitude climbers.^87-89^ Significant slowing in mental processing speed on the Trail Making Test^90^ and specific Time Reaction Tests^85^ alterations have been demonstrated in comparisons of individuals submitted to various levels of hypoxia.

Moderate to severe cognitive decline has been found in 42% of patients (n=203) with COPD and in 14% of controls. Abstract thinking and complex perceptual-motor integration were the more affected domains. Fifty percent of patients presented slowing of motor speed and altered hand coordination.^50^

Some authors consider COPD a model of study for cognitive impairments secondary to chronic hypoxia due to lung disease.^53^ Memory impairment,^49,53,56^ verbal language loss,^53^ attention disturbance,^53,59.62,63.65,66^ dysexecutive syndrome^65,66.69,75^ and difficulties in abstract thinking^53^ were found, while visual attention can be relatively preserved. Other authors argue that there is also visual attention impairment.^54^ A pattern of neuropsychological impairment characterized by verbal tasks and verbal memory deficit was found in 48.5% (n=36) of COPD patients compared to controls with probable Alzheimer’s disease.^53^ In another study, verbal memory profile was assessed in 38.1% (n=42) of patients with COPD. Patients failed memory access and recall tasks.^56^ Low forced expiratory volume of first second (FEV1s) and forced vital capacity (FVC) are predictive parameters of cognitive impairment in COPD.^57^

Recently, cognitive impairment in non hypoxaemic patients has been described. These patients performed significantly worse on the Trail Making Test,^90^ Digit-Span Test (Wechsler Adult Intelligence Scale-III)^90^ and other specific subtests which showed mainly mental processing speed reduction. Memory and cognitive flexibility were relatively preserved. No correlation was found between cognition and worsening in life quality (63). The benefit of prolonged oxygen supplementation therapy has previously been demonstrated.^60^

Comparing studies becomes difficult because of design study variability, sereneness of disease, selection of patients and control groups, and respective study inclusion and exclusion criteria. Other variables such as the use of continuous oxygen therapy, the neuropsychological battery chosen, and treatment prescribed are also confounding factors.

In summary, the majority of both national and international literature on hypoxia cognitive effects in patients with chronic lung disease points to subcortical type mild cognitive impairment with decline in attention, slower mental speed and compromised executive functions.

The expression “subcortical dementia” is attributed to a group of signs and symptoms associated to diseases that involve subcortical structures.^91-92^ Subcortical dementia is characterized by:

1) cognitive slowing (bradyphrenia) with impairment in attention, concentration and executive abilities, including planning and strategy use difficulties, visual-spatial and memory deficit, with the latter affecting data retrieval rather than learning;
2) absence of aphasia, apraxia and agnosia, which constitute classic cortical symptoms and
3) emotional and psychiatric features such as apathy, depression or personality changes.^91^

This syndrome is also called frontal-subcortical dementia, because it can involve lesions in frontal-subcortical pathways or in subcortical structures closely linked to the frontal lobes.^93,94^ Attention and executive circuits involve pre-frontal cortex, thalamus, nucleus *accumbens* and heteromodal cortices (frontal, parietal and occipital) as well as para-limbic associated areas. The main neurotransmitter is acetylcholine, but there are also serotoninergic and dopaminergic pathways. The association between hypoxia and acetylcholine pathways has been the subject of study for two decades, especially in animal models. There is evidence of low acetylcholine concentration in the neocortex, hippocampus, striate nucleus and septal area, as well as dopamine in neocortex and hippocampus, of mice submitted to the same conditions.^95^ This finding could be explained by the proportional reduction in acetylcholine synthesis and other aminoacids due to lower carbohydrate oxidation in mild chronic hypoxia.^95-97^ In addition, the decrease in sodium and potassium ion gradients which occur in chronic hypoxia conditions, jeopardizes acetylcholine transport to neurons, lowering its uptake by the post-synaptic neuron.^98^

In everyday clinical practice, there is an overlapping of cortical and subcortical profiles of deficits and the same can occur for psychiatric symptoms. However, this didactic categorization helps clinicians to distinguish the predominant cognitive-behavioral pattern and thus to reach differential diagnosis. According to previously cited data, COPD^49-75^ and OSAS^76-84^ as well as other systemic diseases, such as cardiac failure^99^ and hepatic insufficiency^100^, can affect cognition. The cognitive syndrome presented varies from predominant subcortical type impairment to overt dementia. Before presenting full dementia, these patients go throughout a transition phase characterized by mild cognitive impairment, in which a decrease in mental speed (bradyphrenia) is frequently the first symptom.^101-103^

The formal current recommendations of the Brazilian Heath Secretariat (104) and Brazilian Society of Tisiology and Pulmonology^105^ for use of prolonged home oxygen supplementation are:

a) PaO2=55 mmHg or SaO2 less than or equal to 88%; or
b) PaCO2=56 to 59 mmHg, or SaO2 less than or equal to 89%, associated to heart failure edema, evidence of *cor pulmonale* or hematocrit level above 56%.

These data must be obtained through arterial blood gas analysis in a rest state while breathing ambient air in a clinically stable patient with the best possible adequate therapy. Formal indication for using these therapies should be questioned and reevaluated in view of study results of cognitive performance enhancement after using continuous oxygen supplementation or continuous positive airway pressure (CPAP) in patients with COPD and OSAS, respectively.

Another relevant issue is the impact of cognitive impairment on adherence to inhaled drugs in patients with COPD. Allen and coworkers had demonstrated that low performance on the MMSE and its intersected pentagon component are significantly associated to worse performance in the ability to learn and retain inhaler techniques.^106-108^ Other executive function and praxis tests were also associated to low adherence in using inhaled medications.^107,109^

Prognostic implications of cognitive impairment in COPD have previously been studied. Worse performance on neuropsychological tests is associated with higher COPD patient mortality.^64,110^ This finding may be explained by two main hypotheses: firstly, COPD patients with worse cognitive performance might be at a more advanced stage of the disease, presenting severe hypoxia which are associated to lower survival rates; secondly these patients may have poor adherence not only to inhaler medication techniques, as stated above, but also to oral and other co-morbidity drugs such as insulin pens.

## Neuroimaging and chronic hypoxia

White matter periventricular and/or subcortical lesions have long been linked to cognitive deficits.^111-114^ These white matter lesions are mainly caused by small artery cerebrovascular disease. The vast majority of these lesions result from cholesterol deposition at the endovascular lining and from its local complications.^115-118^ The cognitive impairment found secondary to small artery cerebrovascular disease can range from mild cognitive impairment to vascular dementia.^118,119^ Nevertheless, two preliminary studies (120, 121) question whether white matter lesions are associated to hypoxic ischemia secondary to pulmonary disease per se. Van Dijk and coworkers (2004) evaluated 1077 non-demented healthy subjects with ages ranging from 60 to 90 years, measured their pulse oxymetry and performed magnetic resonance imaging. These authors concluded that low oxygen saturation and COPD are associated to more severe white matter periventricular lesions. One of the main difficulties found in this kind of research is how to deal with vascular risk factors. More studies are necessary to elucidate this issue.

## Conclusion

Cognitive effects of clinical diseases are given limited importance in congresses and symposia on cognitive impairment and its etiology. Professionals that deal with patients presenting cognitive loss may have a tendency to more frequently suspect degenerative disorders and neglect possible contributions of clinical diseases. Scientists have long restricted their interest in cognitive complications of ischaemic hypoxia to cerebrovascular disease and hypoxic-ischaemic encephalopathy studies both in clinical and basic science research. Experimental models have been based on neonatal hypoxia, post cardiac arrest brain damage and ischemic cerebrovascular disease which are suited to studying brain effects of acute hypoxia. More recently, as mentioned previously, COPD models and possibly idiopathic pulmonary fibrosis models, may help us to broaden our knowledge on cognitive changes secondary to chronic hypoxia and perhaps lead to new insights into diagnosis and treatment.
